# Environmentally friendly fabrication of Ag nanoparticles decorated on g-C_3_N_4_ for enhancing the photodegradation of RhB†

**DOI:** 10.1039/d5na00552c

**Published:** 2025-07-10

**Authors:** Lan Anh Luu Thi, Quoc Tung Trieu, Thi Hue Trinh, Tuyet Mai Nguyen Thi, Cong Tu Nguyen, Tran Thanh Tung, Nguyen Xuan Sang

**Affiliations:** a Faculty Engineering of Physics, Hanoi University of Science and Technology No. 1, Dai Co Viet Street, Hai Ba Trung District 100000 Hanoi Vietnam; b Faculty of Electronics and Telecommunications, Electric Power University No. 235 Hoang Quoc Viet Street Hanoi Vietnam; c School of Chemistry and Life Sciences, Hanoi University of Science and Technology No. 1, Dai Co Viet Street, Hai Ba Trung District 100000 Hanoi Vietnam; d The University of Adelaide, School of Chemical Engineering Adelaide SA 5005 Australia; e Atomic Molecular and Optical Physics Research Group, Institute for Advanced Study in Technology, Ton Duc Thang University Ho Chi Minh City Vietnam nguyenxuansang@tdtu.edu.vn; f Faculty of Electrical and Electronics Engineering, Ton Duc Thang University Ho Chi Minh City Vietnam

## Abstract

This work presents an eco-friendly method for the preparation of a silver nanoparticle (AgNP) decorated g-C_3_N_4_ nanocomposite using purple leaf extract as a green reduction agent. The photocatalytic performance of the resulting nanocomposite was studied through the degradation of rhodamine B (RhB) dye pollution in an aqueous solution. X-ray diffraction analysis of powder samples revealed the coexistence of face-centered cubic AgNP crystalline and g-C_3_N_4_ structures, with a slight shift in the dominant diffraction peak (002) of g-C_3_N_4_, indicating successful incorporation of AgNPs. Optical analysis showed a reduction in the bandgap of the nanocomposite compared with that of the pure g-C_3_N_4_ sample. The photocatalytic ability of the nanocomposites was tested through the degradation of RhB dye, which was significantly enhanced by the presence of AgNPs, achieving a maximum degradation efficiency of approximately 95.3% after only 75 minutes of irradiation using the Ag@g-C_3_N_4_ nanocomposite with 7 wt% AgNPs as a photocatalyst. This enhancement is attributed to the efficient charge carrier separation and suppressed recombination rate at the photocatalyst interfaces.

## Abstract 2

This work presents an eco-friendly method for the preparation of a silver nanoparticle (AgNP) decorated g-C_3_N_4_ nanocomposite using purple leaf extract as a green reduction agent.

## Graphical abstract

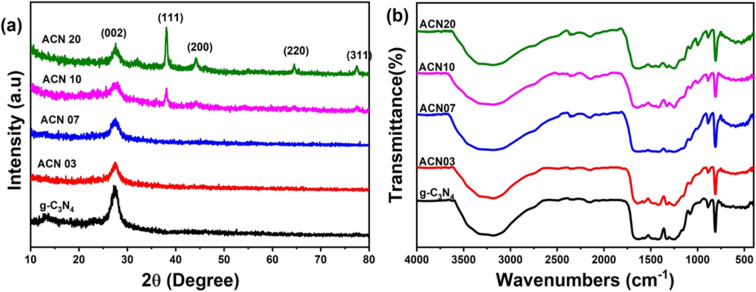

## 1. Introduction

Organic dyes, especially synthetic organic dyestuffs, are widely used across various industries, including textile dyeing, printing, leather processing, papermaking, and cosmetics, to meet both individual and societal demands.^1^ Most dyes possess complex structures and high molecular weights, are water soluble and are barely biodegradable, thus posing a serious threat to the environment.^2^ Currently, environmental treatment in general and wastewater treatment in particular have become a hotspot not only in basic research but also in applied research, and increasing research focuses on removing pollutants from water using new technologies.^3–8^ For the survival of humans and other living organisms, it is important to decompose dyes in an environmentally friendly and highly efficient way. Among the various approaches explored, photocatalysis has gained considerable attention. In this context, recently, graphitic carbon nitride (g-C_3_N_4_) has emerged as a potential photocatalyst that has been extensively studied^9–11^ due to its unique properties, such as high thermal and chemical stabilities, abundant and low-cost building elements, environmental friendliness, and especially a suitable electronic structure with band edges straddling the water redox potentials, making it highly suitable for application in photocatalysis.^12–14^ g-C_3_N_4_ is a polymer semiconductor that has been widely used in photocatalytic research because of its outstanding activity for various catalytic reactions, such as the decomposition of organic pollutants,^15^ producing H_2_ and O_2_ by splitting water^16^ and reducing CO into organic fuels.^17^ However, the high recombination rate of photogenerated electron–hole pairs and limited active sites limit the applications of this material.^18^

Various methods, including increasing the surface area, reducing the electron–hole recombination rate and extending the visible light absorption region to longer wavelengths, are among the studies that have been carried out to improve the photocatalytic activity of g-C_3_N_4_. One of the effective solutions to separate the electron–hole pairs and prevent their recombination is to create composite materials using g-C_3_N_4_ and other metal nanoparticles. This hybridization significantly reduces electron–hole recombination as noble metal nanoparticles can serve as efficient electron acceptors due to their strong surface plasmon resonance effect.^19,20^ To enhance the photocatalytic activity, other semiconductor oxides such as TiO_2_ can also be modified with Ag to reduce contaminants in an aqueous phase.^21^ The doping, hybridization, modification or compositing of Ag with g-C_3_N_4_ may change the conductivity of the composite material compared to that of pure g-C_3_N_4_ due to the reduction of the energy barrier for the transfer of electrons. Pham *et al.* reported the fabrication of Ag/g-C_3_N_4_ material *via* a photoreduction method. The 20% Ag/g-C_3_N_4_ sample provides the highest photocatalytic efficiency of NO decomposition and reaches 80% under visible light irradiation.^22^ Dong Liang *et al.* prepared Ag/g-C_3_N_4_ for which the g-C_3_N_4_ was synthesized by urea pyrolysis at 550 °C with a heating rate of 15 °C min^−1^ for 3 h. The obtained results showed that the photocatalytic nitration of pharmaceutical intermediate halo-nitro-phenol, using various bromophenols and nitrites as raw materials, was significantly improved when using Ag/g-C_3_N_4_ composite as the photocatalyst compared with pure g-C_3_N_4_.^23^ In another work, the Ag/g-C_3_N_4_ composite was synthesized *via* polymerization and the silver mirror reaction. In the sample surveys, the sample containing 5% Ag had the highest hydrogen production rate which was 39 times higher than that of bulk g-C_3_N_4_ with a value of 568.9 μmol g^−1^ h^−1^.^24^ Hai Zhu *et al.* used an ultrasound-assisted method to prepare Ag/g-C_3_N_4_ catalysts for photocatalytic water splitting. The results showed that the optimal synthesis conditions were 60 W ultrasonic power for 35 s (residence time). The sample showing the best photocatalytic activity for the water splitting reaction was the 7 wt% Ag/g-C_3_N_4_ sample, which had a reaction rate constant 2.76 times higher than that of pristine g-C_3_N_4_.^25^

In this study, Ag was selected to investigate its interaction with g-C_3_N_4_ and its influence on the structural, physical and chemical properties and photocatalytic ability of g-C_3_N_4_. The photocatalytic activity was examined through the degradation of RhB dye. In addition, the effects of reaction parameters on the photocatalytic performance and the photocatalytic enhancement mechanism were systematically evaluated.

## 2. Experimental

### 2.1 Chemical materials

Urea was purchased from Sigma and silver nitrate was purchased from Xilong Scientific Co., Ltd (China). Double distilled water (Aquatron A4000D) was used in all experiments. All chemicals were used as received without further purification.

### 2.2 Synthesis of Ag@g-C_3_N_4_ nanocomposites

The g-C_3_N_4_ was synthesized from urea using a thermal condensation method, as follows. 40 g of urea was placed in a crucible with a lid, then the sample was annealed at 500 °C for 2 h with a heating rate of 5 °C min^−1^. The product achieved is g-C_3_N_4_ in light yellow bulk form. Purple leaves were extracted according to the procedure described in the ref. 26.

**Scheme 1:**
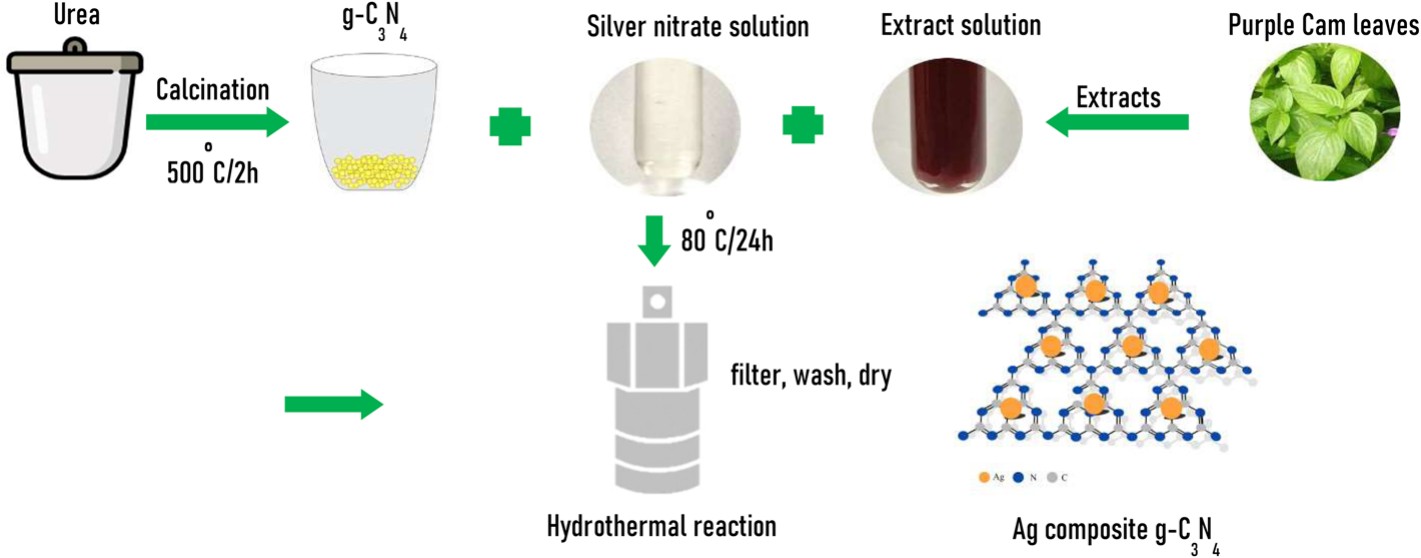
Schematic of the preparation process of Ag@g-C_3_N_4_ nanocomposites.

For the synthesis of Ag@g-C_3_N_4_ nanocomposites, predetermined amounts of AgNO_3_ and g-C_3_N_4_ were dissolved into 50 ml of purple leaf extract under the effect of magnetic stirring for about 60 minutes to obtain 50 ml of precursor suspension solution. This solution was transferred to a 100 ml hydrothermal autoclave. The hydrothermal reaction was performed at 80 °C for 24 h. The suspension obtained after the reaction was washed several times with distilled water to remove the by-products, and the samples were dried at 80 °C for 24 h. The experimental scheme is illustrated in Scheme 1. The Ag@g-C_3_N_4_ nanocomposites were designated according to their Ag contents as summarized in Table 1. The obtained samples are denoted as g-C_3_N_4_, ACN03, ACN07, ACN10 and ACN20, respectively, corresponding to the Ag contents in the synthesized samples of 3, 7, 10, and 20 weight percentage.

**Table 1:** Sample name and the corresponding mass percentage of silver used in the nanocomposites

Sample name	Weight percent of Ag (wt%)	Weight of g-C_3_N_4_ (g)	Weight of Ag (g)
g-C_3_N_4_	0	0.300	0.000
ACN03	3	0.291	0.009
ACN07	7	0.279	0.021
ACN10	10	0.270	0.030
ACN20	20	0.240	0.060

### 2.3 Analysis

The structural characteristics of the nanocomposites were analyzed from the XRD data of the powder samples using an X'pert Pro (PANalytical) MPD system with CuKα radiation (*λ* = 1.54065 Å), a scanning speed of 0.03° per 2 s, and 2*θ* values from 10° to 80°. The vibrational characteristics of the functional groups were found from the FTIR spectra and measured on a FT/IR-4600 TypeA system (JASCO) with wavenumbers from 500 cm^−1^ to 4000 cm^−1^. The surface morphology of the nanocomposites was observed through FESEM (Hitachi S4800) and TEM image analysis (JEM1010-7600F, JEOL). The reflectance spectra of the nanocomposites were also examined using a JASCO V-750 system with an ISV-922 60 mm integrating sphere.

### 2.4 Photocatalytic studies

The photocatalytic properties of the nanocomposite samples were evaluated by their ability to degrade RhB dye under visible light irradiation. The assessment was conducted with a photocatalytic reaction chamber with an LED lamp (Highbay 150 W, SH-HB2-150 W, *λ* ≥ 450 nm). The details are as follows: 20 mg of Ag@g-C_3_N_4_ nanocomposite was well-dispersed into 50 ml of 10 ppm RhB solution. Then, the mixture was stirred in the dark for about 30 min to establish the adsorption and desorption equilibrium between the dye molecules and the photocatalyst surface. Next, the solution was illuminated by LED light. At certain time intervals, about 3 ml of the solution was taken out and centrifuged to separate the catalyst. The absorbance of the solution was measured using a UV-vis absorption spectrophotometer (Carry 100 double beam UV/vis spectrophotometer, VARIAN). In order to elucidate the photocatalytic degradation mechanism and identify the active species involved, radical scavenger experiments were conducted. Specific trapping agents such as isopropanol (1 mM), benzoquinone (1 mM) and sodium oxalate (1 mM) were individually added to the solution.

## 3. Results and discussion

### 3.1 Characterizations of the Ag@g-C_3_N_4_ (ACN) nanocomposite samples

**Fig. 1:**
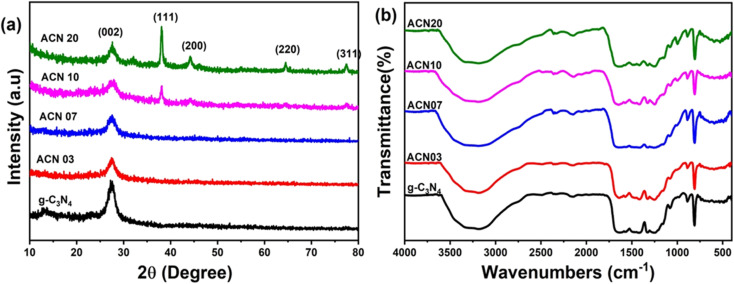
(a) XRD patterns and (b) FT-IR spectra of ACN nanocomposite samples.

The XRD patterns of the Ag@g-C_3_N_4_ nanocomposite samples measured in the 2*θ* angle range of 10°–80° are shown in Fig. 1a. In the as-prepared g-C_3_N_4_ sample, three diffraction peaks are observed at approximately 13.00°, 24.93° and 27.65°, attributed to the (100), (101) and (002) diffraction planes, respectively. These peaks are associated with the hexagonal phase of graphitic carbon nitride corresponding to the standard JCPDS card no. 87-1526.^27,28^ In the Ag@g-C_3_N_4_ nanocomposite samples, additional diffraction peaks appear as the silver content increases, particularly up to 20 wt%. These new peaks are attributed to the FCC crystalline structure of silver, with the characteristic reflections at 2*θ* values of 38.1°, 44.3°, 64.4°, and 77.5° corresponding to the (111), (200), (220), and (311) planes, respectively.^26^

Furthermore, the characteristic diffraction peak position (002) of the g-C_3_N_4_ nanocomposite samples was observed to gradually shift slightly toward lower 2*θ* values when the amount of Ag increased. This may indicate the influence of Ag nanoparticles on the crystal structure of g-C_3_N_4_, potentially due to lattice distortion or interfacial interactions.

The vibrations of the organic functional groups in ACN nanocomposite samples were studied using FTIR spectroscopy, as shown in Fig. 1b. The absorption peaks observed at wavenumbers 1640, 1564, 1413, 1326, and 1247 cm^−1^ are attributed to the typical stretching vibrations of CN heterojunctions.^26,29^ The vibration modes at wavenumbers 810 and 3169 cm^−1^ are due to the stretching vibrations of the triazine units and –NH, respectively. The wavenumber range from 3600 cm^−1^ to 3000 cm^−1^ corresponds to the O–H stretching vibration and a small amount of absorbed H_2_O.^30,31^ Furthermore, it was found that the shape of the FTIR spectra in all ACN samples remained almost unchanged.

**Fig. 2:**
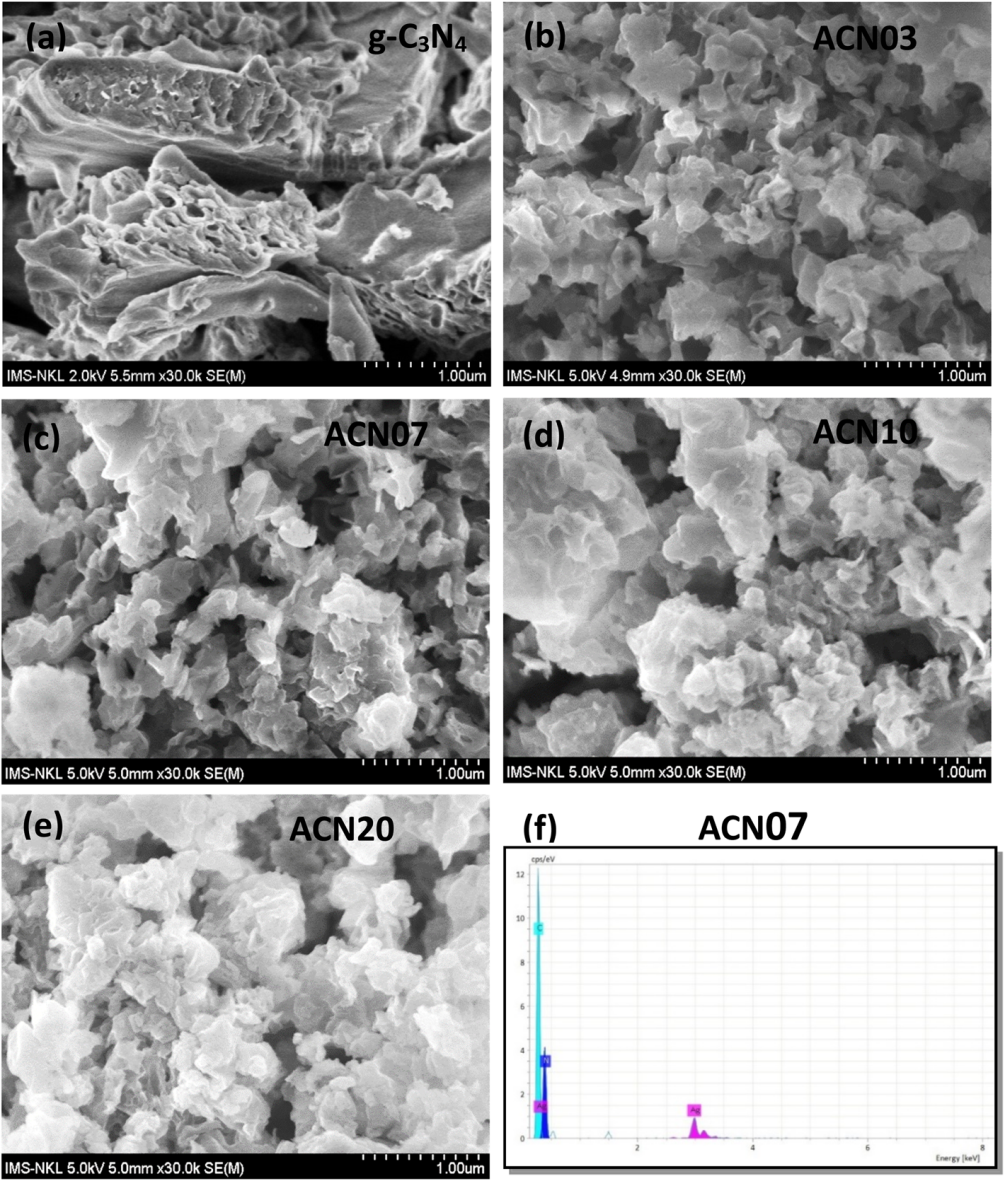
FESEM images of the ACN nanocomposite samples: (a) g-C_3_N_4_, (b) ACN03, (c) ACN07, (d) ACN10, and (e) ACN20. (f) EDX spectrum of the ACN07 sample.

Fig. 2 presents the FESEM images of the ACN nanocomposite samples. The as-prepared g-C_3_N_4_ sample exhibits a morphology of overlapping layered sheets, resembling a graphite-like structure. When silver nanoparticles decorate the g-C_3_N_4_ surface, a noticeable change in surface morphology is observed. As the amount of AgNPs in the nanocomposite sample increased, a progressive accumulation of Ag NPs on the g-C_3_N_4_ surface was evident, forming clusters of particles (samples ACN03, ACN07, ACN10 and ACN20).

To determine the silver content in the sample and compare it with theoretical calculations, the atomic compositions of the g-C_3_N_4_ and ACN07 samples were analyzed by energy dispersive X-ray spectroscopy (EDX). Fig. 2f shows the EDX spectrum results of the ACN07 sample. In the EDX spectrum, the characteristic peaks of the elements C, N and Ag appear, proving that in sample ACN07, there are elements C, N and Ag. Hence, the ACN nanocomposites possess high purity.

**Fig. 3:**
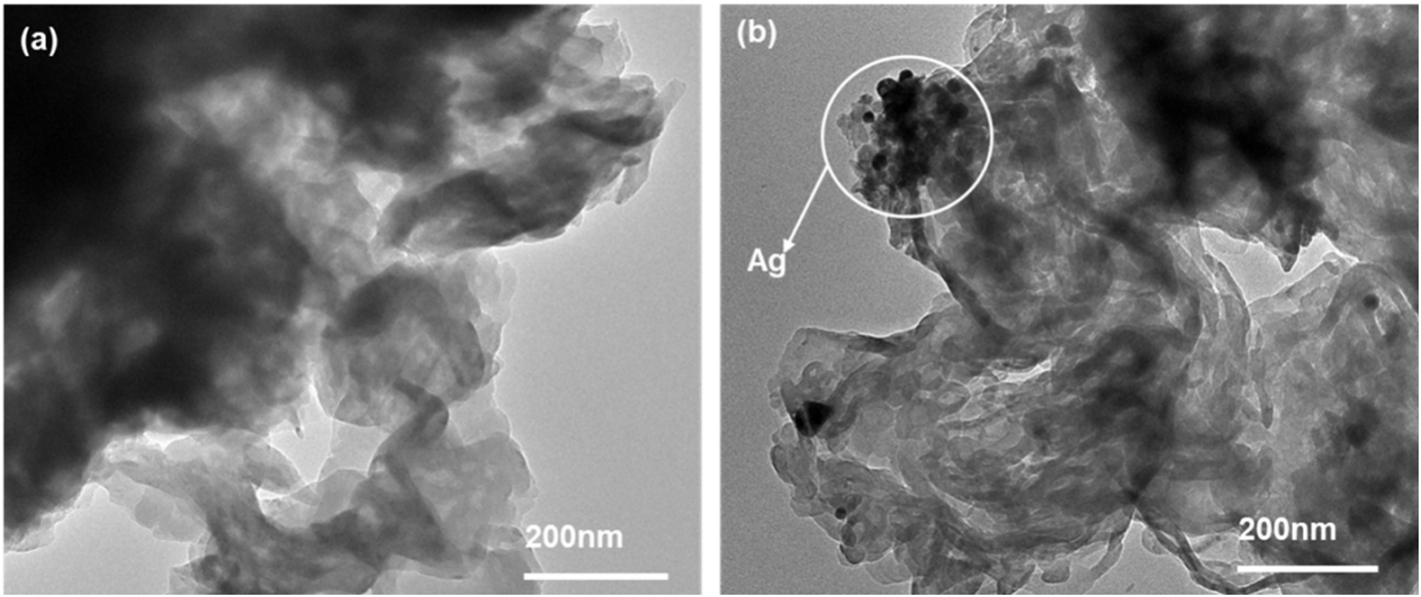
TEM images of (a) g-C_3_N_4_ and (b) ACN07.

To further analyze the surface morphology of the composites, transmission electron microscopy (TEM) images of the CN and ACN07 samples were obtained and are shown in Fig. 3. The CN sample exhibits an uneven, layered structure with an irregular morphology. In contrast, the ACN07 sample shows the presence of small, dispersed particles which are attributed to the incorporation of Ag NPs into the g-C_3_N_4_ matrix.

**Fig. 4:**
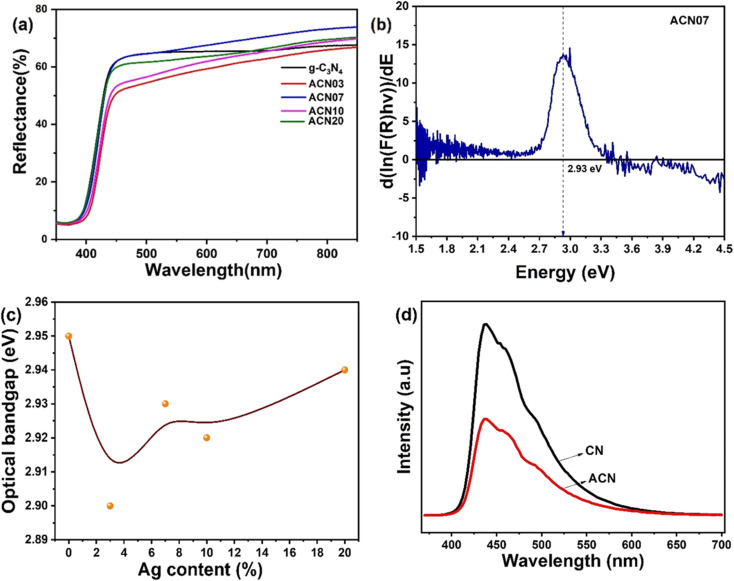
(a) Reflectance spectra. (b) Optical bandgaps of the ACN07 nanocomposite samples obtained by the first-derivative method. (c) Graph of optical bandgap *vs.* AgNPs content. (d) PL spectra of the ACN nanocomposite samples.

Fig. 4a shows the reflectance spectra of the ACN nanocomposite samples. It is observed that the reflectance of the ACN nanocomposite samples remarkably increases when incorporating Ag nanoparticles. Moreover, the reflectance edge also shifts to a longer wavelength when the content of Ag increases, which implies more absorbance of visible light. The reason for this redshift is believed to be due to the plasmonic properties of silver nanoparticles in nanocomposite samples, which effectively absorb most of the light in the visible and infrared regions.

To further study the optical properties of the nanocomposite samples and evaluate their efficiency in visible-light-induced applications such as photocatalysis under solar irradiation, the optical bandgaps of the nanocomposite samples were extracted from the derivative method based on the Kubelka–Munk equation^32,33^ and expressed as1
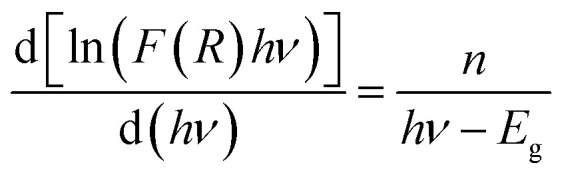
where 
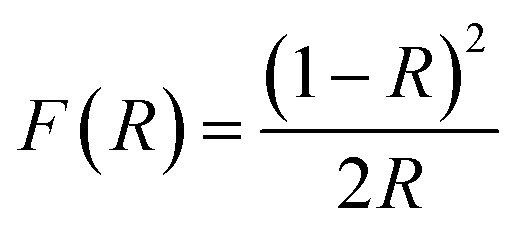
 and 
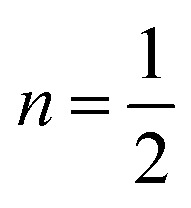
 or 2 for direct or indirect allowed recombination, respectively.^34^

The first derivative induced optical bandgaps of ACN nanocomposite are shown in Fig. 4b, and Fig. 4c shows that the optical band gap energy decreases with increasing silver content. This result might originate from the increase of absorbance due to the plasmonic resonance properties of Ag in the nanocomposites.

The PL fluorescence spectra of g-C_3_N_4_ and ACN07 samples, shown in Fig. 4d, indicated that the maximum emission of g-C_3_N_4_ (CN) is ∼435 nm and it has a higher intensity than the ACN07 sample, consistent with the results observed in the UV vis absorption spectra. This proves that the regeneration rate of photogenerated carriers in g-C_3_N_4_ is faster. In contrast, in the ACN07 sample, the recombination rate is slower or the lifetimes of electrons and holes are longer. This means that the photocatalytic ability is enhanced, because the longer the electrons and holes exist, the more carriers participate in the toxic compound decomposition reactions, leading to higher treatment efficiency.

### 3.2 Photocatalytic activity of the Ag@g-C_3_N_4_ (ACN) nanocomposite samples in decomposing rhodamine B dye

**Fig. 5:**
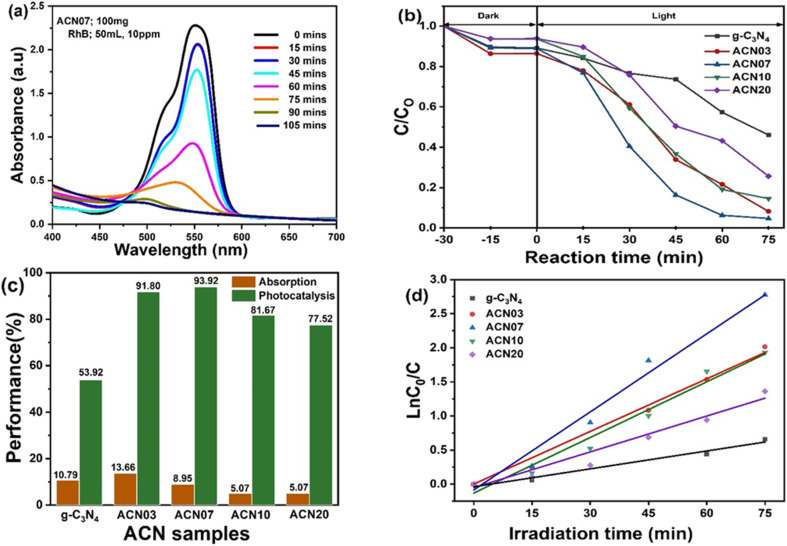
(a) Sequence of RhB photodegradation on the ACN07 nanocomposite sample under visible-light irradiation. (b) Photodegradation of RhB at various reaction times under dark and visible light conditions. (c) Photodegradation performances for RhB on the ACN nanocomposite samples. (d) First-order kinetic curves of RhB decomposition on the ACN nanocomposite samples under visible-light irradiation.

The decrease in RhB concentration over the photocatalytic reaction time is shown in Fig. 5a. The dominant peak located at ∼550 nm is assigned to the absorption peak of RhB.^35^ As the reaction time progressed, the intensity of the RhB adsorption peak gradually decreased, indicating that the concentration of RhB in the solution gradually decreased. In other words, RhB was decomposed by photocatalytic reaction using ACN nanocomposite as the catalyst. Furthermore, the dominant peak was slightly blue-shifted by the time of irradiation corresponding to the color of the solution changing from pink-red to greenish-yellow. Hence, RhB was decomposed into intermediate substances.^36–38^

The graphs of RhB concentration dependence on reaction time are shown in Fig. 5b. Over 30 minutes, during adsorption–desorption equilibrium in the dark, RhB concentration decreased by about 5–15%. After 75 minutes of illumination, the RhB was almost completely decomposed. The ACN07 sample gives the fastest decomposition of RhB solution, with an efficiency of 93.9%, followed by samples ACN00 and ACN03, which are slightly lower at 92.2% and 91.7%. The ACN10 and ACN20 samples showed relatively low efficiencies, with 81.67% and 77.52% (Fig. 5c), respectively. Thus, the photocatalytic efficiency of the Ag@g-C_3_N_4_ nanocomposite sample depends on the Ag ratio.

The photocatalytic reaction rate is expressed through the Langmuir–Hinshelwood kinetic equation2
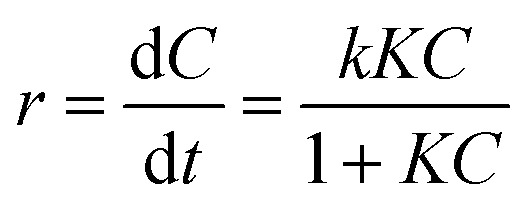
where *r* is the reaction rate, *C* is the reactant concentration, *t* is the illumination time, *k* is a constant and *K* is the absorbance coefficient of the reactant. When the concentration of the substance is relatively small, the equation can be simplified to3
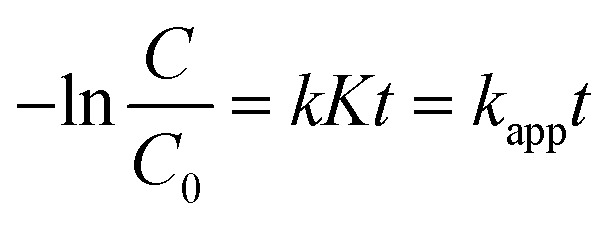
where *k*_app_ is the rate coefficient and is obtained from the slope of the linear function graph of the equation.

Fig. 5d shows the interpolated linear function according to the experimental data of each ACN nanocomposite sample and the *R*^2^ coefficient. Table 2 gives the *k*_app_ rate constants and *R*^2^ constants of the ACN*x* nanocomposite samples. The *R*^2^ coefficients of all nanocomposite samples are greater than 0.97, so the Langmuir–Hinshelwood model relatively appropriately reflects the photocatalytic process for RhB of the ACN nanocomposite samples. The ACN07 sample has the best photocatalytic ability (*k* = 0.039 min^−1^). It is possible that silver nanoparticles in nanocomposites act not only as an electron pool but also to capture the photoinduced electrons.

**Table 2:** The activity, *k*_app_, TON and TOF of ACN*x* nanocomposite samples

Sample	The content of Ag (wt%)	Activity (%)	*k*_app_ (min^−1^)	*R* ^2^	TON_Ag_	TOF_Ag_ × 10^−4^ (min^−1^)
g-C_3_N_4_	0	53.92	0.009	0.986	—	—
ACN03	3	91.80	0.033	0.977	0.231	30.76
ACN07	7	93.92	0.039	0.970	0.101	13.49
ACN10	10	81.67	0.027	0.976	0.062	8.21
ACN20	20	77.52	0.019	0.980	0.029	3.90

In the catalytic system, TON and TOF are two important parameters used to evaluate the lifetime and performance of a catalyst.^39^ Therefore, TOF per unit amount of Ag NPs (Table 2) was used to evaluate the effect of Ag doping on the material properties. The ACN07 sample has the highest TOF of 30.76 min^−1^. When the Ag doping amount increases, the TOF decreases. This phenomenon occurs because when Ag increases, the amount of metal on the surface increases but its dispersion on the surface decreases. However, the opposite TON and TOF values also indicate that ACN has higher photocatalytic activity than pure g-C_3_N_4_. Therefore, there was a beneficial interaction between Ag and the g-C_3_N_4_ matrix. For details of the TON and TOF calculations, please see the ESI.†

The catalyst dosage also affects the dye decomposition efficiency. If the catalyst dosage is low, it is not enough to create active centers for the dye adsorption process on the catalyst surface, so the dye decomposition efficiency will decrease. If the catalyst dosage is too high, it will block the incident light and reduce light absorption, leading to a decrease in the ability to trap light and preventing the generation of photogenerated electrons and holes, so the dye decomposition efficiency will also decrease. Therefore, an optimal balance between RhB adsorption capacity and photon collection capacity will be achieved with the optimal catalyst dosage, and the efficiency will be optimized.

**Fig. 6:**
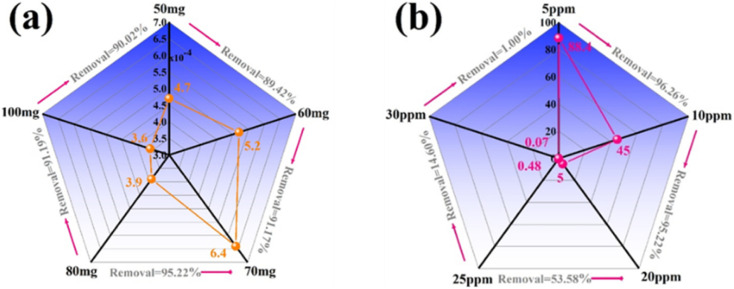
Effects of (a) the dosage of ACN07 and (b) concentration of RhB solution on photocatalytic activity.

Fig. 6a presents the degradation of RhB using the ACN07 nanocomposite at different dosages (50–100 mg). The catalytic reaction rate was significantly enhanced with the dosage of catalyst. To evaluate the effect of dosage on the catalytic performance, the ratio of reaction rate to dosage was calculated. The performance ranking was as follows: 70 mg > 60 mg > 50 mg > 80 mg > 100 mg. Thus, the best sample dosage was 70 mg. Similarly, the RhB degradation reaction rate was affected by the concentration of RhB used (Fig. 6b). At low RhB concentration (5 ppm), the degradation rate of RhB increased significantly compared to that at high concentration (30 ppm), because when the RhB concentration is too high, the adsorption of RhB onto the catalyst decreases. The optimal RhB concentration in this study was 10 ppm.

**Fig. 7:**
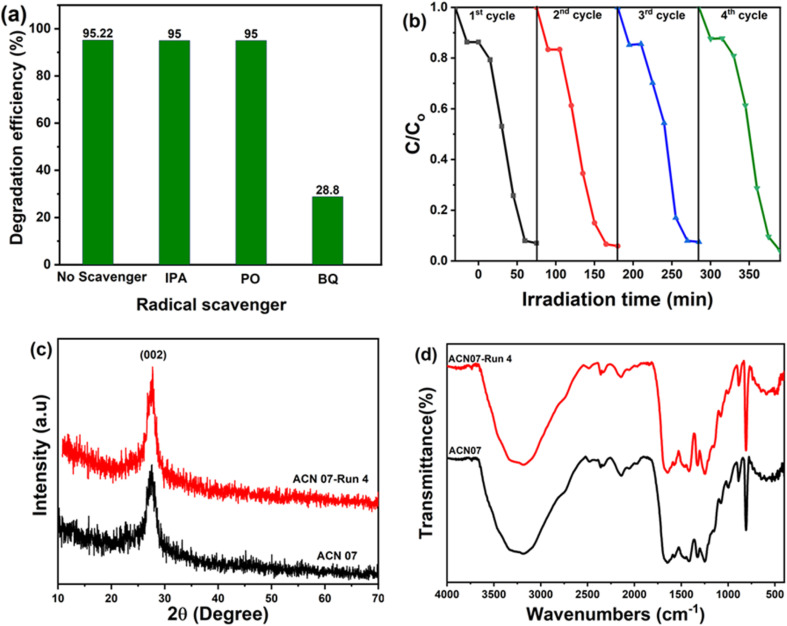
(a) Effect of scavengers. (b) Cycling runs, (c) XRD patterns and (d) FT-IR spectra of the ACN07 sample initially and after the fourth cycle.

The photocatalytic performance of RhB decolourization largely depends on the generation of strong chemically active radicals during the reaction process. The primary active species typically considered include hydroxyl radicals (˙OH), superoxide radicals (˙O_2_^−^), and photogenerated holes (h^+^). In this work, to study the effects of chemically active radicals, three agents were used: benzoquinone (BQ), isopropanol (IPA), and sodium oxalate (NO) for the radicals superoxide (*O_2_^−^), hydroxyl (*OH^−^) and hole (h^+^), respectively. In each experiment, 1 ml of the agent with a 1 mM concentration of the respective scavenger was added into a beaker containing 50 ml of 10 ppm RhB solution containing 0.07 g ACN07. The corresponding decolourization efficiencies in the presence of the agents are shown in Fig. 7a.

The role of reactive species in the photocatalytic degradation of RhB was further investigated through radical scavenging experiments. The addition of isopropanol has little effect on the reaction rate. Thus, it can be confirmed that the hydroxyl radical (*OH^−^) is not essential in the decomposition process of RhB dye. Interestingly, in the presence of the NO scavenger, the photocatalytic activity of the Ag@g-C_3_N_4_ nanocomposite sample increases, so h^+^ is not the active substance in this photodegradation process. In the presence of BQ, the reaction rate decreased the most. At the same time, it was observed that the peak shift phenomenon occurred relatively little, but the spectrum gradually increased with illumination time. The spectral enhancement becomes stronger when moving towards short wavelengths. The photodegradation of the RhB dye was significantly inhibited in the presence of BQ, demonstrating that O_2_^−^ plays a major role in this process.

These results demonstrate that h^+^ and especially O_2_^−^ are the main reactants in the photocatalytic RhB decomposition. The reusability of the Ag@g-C_3_N_4_ photocatalyst was evaluated by four consecutive photocatalytic runs, as seen in Fig. 7b. For each recycling, the photocatalytic material was filtered using a vacuum system to collect the residue. The resulting residue was dried in a dryer at 80 °C for 24 h. Then, the material was ground and photocatalyzed under similar conditions (material weight 0.07 g, RhB dye volume 50 ml at 10 ppm concentration).

RhB photodegradation efficiency was almost complete in each cycle. Therefore, there was no significant loss of catalytic activity even after 4 cycles. Therefore, the Ag@g-C_3_N_4_ nanocomposite is reusable.

Fig. 7c and d show the XRD and FTIR results of the ACN07 sample initially and after the cycling. It is easy to see that the diffraction peaks of the ACN07 sample hardly change after 4 cycles (Fig. 7c). This means that the ACN07 sample has good repeatability and stability. The FTIR measurement results of this sample are consistent with the XRD measurement results.

**Fig. 8:**
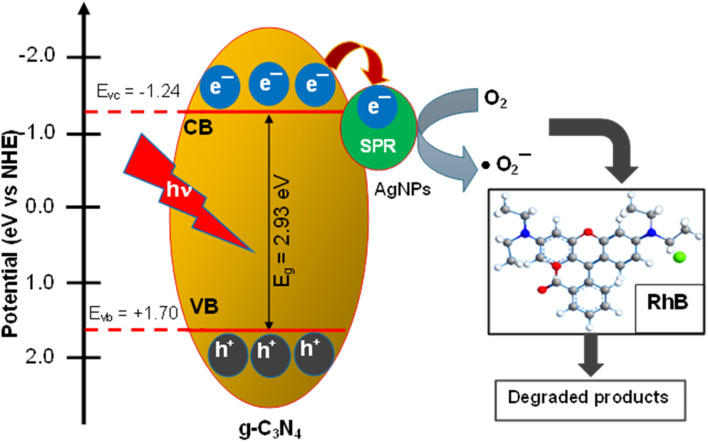
Proposed mechanism for the improved photocatalytic behaviour toward RhB over the ACN composite under LED illumination.

The photocatalytic enhancement mechanism of RhB degradation by ACN nanocomposite can be described as shown in Fig. 8. The *E*_VB_ and *E*_CB_ values of the Ag@g-C_3_N_4_ nanocomposites can be calculated by the equations^40^4
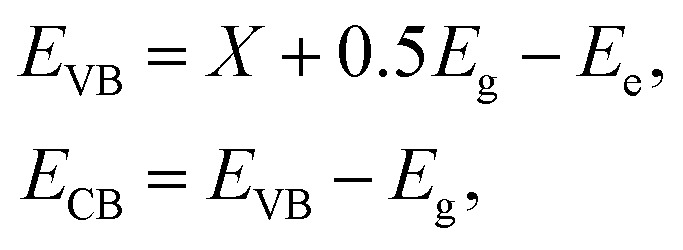
where *E*_VB_ is the valence band potential, *E*_CB_ is the conduction band potential, *X* is the absolute electronegativity of the semiconductor and *E*_e_ is the energy of free electrons on the hydrogen scale (*E*_e_ = 4.5 eV). The *X* value for g-C_3_N_4_ is 4.73 eV. *E*_g_ is the optical band gap of Ag@g-C_3_N_4_. In this case, *E*_g_ = 2.93 eV (ACN07).

The values of *E*_CB_ and *E*_VB_ for g-C_3_N_4_ are estimated to be −1.24 eV and 1.70 eV, respectively.

The reaction process is described in eqn (5)–(8) and is detailed as follows. Under the irradiation of visible light, only g-C_3_N_4_ absorbs visible light and is excited. Then, the electrons in the VB can be transferred to the CB of g-C_3_N_4_ to generate photogenerated electron pairs. Due to the SPR effect of silver in the form of nanoparticles, the generation rate of e–h pairs of g-C_3_N_4_ increases.^41,42^ Finally, the ˙O_2_^−^ species photodegraded RhB into H_2_O and CO_2_ as follows.

5 g-C_3_N_4_ + *hν* → g-C_3_N_4_(e^−^ + h^+^)

6 g-C_3_N_4_(e^−^) → AgNPs(e^−^)

7 AgNPs@g-C_3_N_4_(e^−^) + O_2_ → *O_2_^−^

8 *O_2_^−^ + RhB → CO_2_ + H_2_O

Table 3 summarizes the RhB degradation performance of the ACN07 sample compared with some published results. ACN07 has a very good RhB degradation efficiency of up to 95% in 75 min. Moreover, the reaction can be carried out with an LED lamp (150 W, *λ* ≥ 450 nm). The sample has high stability after 4 cycles. The results indicate the applicability of ACN samples in wastewater treatment.

**Table 3:** Photocatalytic degradation of the RhB dye under visible light

Photocatalyst	Light source	Dosage (mg)	Dye concentration (ppm)	Volume of dye (ml)	Reaction time (min)	Removal efficiency (%)	Ref.
Ag-(P/CNNS)	Xe lamp 300 W	25	10	50	240	98	43
a-AgSiO/CNNS-500	Xe lamp 500 W	50	10	50	150	95	44
Ag@g-C_3_N_4_ NSs	Xe lamp 400 W	2	10	20	250	89	45
0.02Ag/g-C_3_N_4_	100 W	20	5	100	25	97	46
AgI/g-C_3_N_4_	Osram, 125 W	50	5	50	120	80	47
ACN07	LED lamp, 150 W	70	10	50	75	95	This work

## 4. Conclusion

In summary, AgNPs@g-C_3_N_4_ nanocomposites were successfully synthesized through a thermal hydrolysis method at low temperatures using purple leaf extract as a reducing agent and solvent. The resulting nanocomposite materials showed significantly enhanced photocatalytic activity compared to pure g-C_3_N_4_, primarily due to the surface plasma resonance (SPR) effect of the Ag NPs, and a reduced recombination rate of the photogenerated e^−^–h^+^ pairs. Among the series of photocatalysts, the ACN07 sample demonstrated the highest photocatalytic efficiency, achieving a RhB degradation rate of 95.3% under visible light irradiation. The optimal parameters for the photocatalytic process were identified as 0.7 g of ACN07 in 50 ml of 10 ppm RhB solution irradiated for 75 minutes of illumination using an LED lamp, and the photocatalyst remained stable after 4 cycles. The experiment also showed that the main agent enhancing the photocatalytic activity of the ACN nanocomposite is the super oxygen free radical.

## Author contributions

Nguyen Xuan Sang: writing – original draft, writing – review & editing, visualization, validation, supervision, and conceptualization. Quoc Tung Trieu: data curation, software, methodology, investigation, and formal analysis. Thi Hue Trinh: data curation, methodology, investigation, and formal analysis. Thi Tuyet Mai Nguyen: validation, resources, investigation, formal analysis, and conceptualization. Cong Tu Nguyen: writing – original draft, writing – review & editing, visualization, validation, supervision, and conceptualization. Tran Thanh Tung: writing – review & editing, visualization, validation, and conceptualization. Thi Lan Anh Luu: supervision, methodology, writing – original draft, writing – review & editing, conceptualization, and funding acquisition.

## Conflicts of interest

The authors declare that they have no known competing financial interests or personal relationships that could have influenced the work reported in this paper.

## Supplementary Material

NA-OLF-D5NA00552C-s001

## Data Availability

The data supporting this article have been included as part of the ESI.†
