# Supplementary material for: Environmentally friendly fabrication of Ag nanoparticles decorated on g-C3N4 for enhancing the photodegradation of RhB
Source: Nanoscale Adv. 2025 Jul 10;7(17):5250–61. doi: 10.1039/d5na00552c (PMC12278500; doi:10.1039/d5na00552c)
Supplement: NA-007-D5NA00552C-s001 [file NA-007-D5NA00552C-s001.pdf]

Supplementary Information (SI) for Nanoscale Advances.
This journal is © The Royal Society of Chemistry 2025

Supporting Information

Environmentally eco-friendly fabrication of Ag nanoparticles decorated on g-C_3N_4 for enhancing the photodegradation of RhB

Lan Anh Luu Thi[1], Quoc Tung Trieu[1], Thi Hue Trinh[2], Tuyet Mai Nguyen Thi[3], Cong Tu Nguyen[1], Tran Thanh Tung[4] and Nguyen Xuan Sang[5,6*]

[1]*Faculty Engineering of Physics, Hanoi University of Science and Technology, No 1, Dai Co Viet Street, Hai Ba Trung district, 100000 Hanoi, Viet Nam*
[2]*Faculty of Electronics and Telecommunications, Electric Power University, No. 235 Hoang Quoc Viet Street, Hanoi, Vietnam*
[3]*School of Chemistry and Life Sciences, Hanoi University of Science and Technology, No 1, Dai Co Viet Street, Hai Ba Trung district, 100000 Hanoi, Viet Nam*
[4]*The University of Adelaide, School of Chemical Engineering, Adelaide, SA 5005, Australia*
[5]*Atomic Molecular and Optical Physics Research Group, Institute for Advanced Study in Technology, Ton Duc Thang University, Ho Chi Minh City, Vietnam*
[6]*Faculty of Electrical and Electronics Engineering, Ton Duc Thang University, Ho Chi Minh City, Vietnam*

[*]*Corresponding authors: nguyenxuansang@tdtu.edu.vn*

Text S1. Calculation of TOF and TON

For $AgNO_3$ of Ag@g-C_3N_4:

Molecular weight of $AgNO_3$ in Ag@g-C_3N_4 is 169.87

Atomic weight of Ag is 107.87

The 169.87g $AgNO_3$ contains 107.87g Ag

So 50mg of $AgNO_3$ contains

$$\frac{0.05 \times 107.87}{169.87} = 0.027\text{g of Ag}$$

One mole of $Ag@g\text{-}C_3N_4$ solution contains 169.87g $AgNO_3$, dissolved it in 1L.

Thus, in 1L of $Ag@g\text{-}C_3N_4$ solution there is 107.87g Ag

0.027 g of Ag is equivalent to

$\frac{0.027}{107.87} = 2.5 \times 10^{-4}$ moles of Ag; number of active sites

Degradation data of substrate RhB under visible light irradiation were shown in (Figure 5).

479g of Rhodamine is dissolved in 1L of solution to obtain 1M of solution 10mg of Rhodamine dissolved in 1L of solution, equivalent to:

$\frac{0.01 \times 1000 \times 1}{479 \times 1000} = 2.097\times10^{-5}$ moles

For ACN07 sample:

93.92% of RhB is degraded under Visible light with $Ag@g\text{-}C_3N_4$ as catalyst

93.92% of 2.097×10^{-5} moles are 1.9695×10^{-5}moles. More specifically 1.9695×10^{-5} moles were degraded in 75 min time period

$$\text{TON} = \frac{(Number\ of\ moles\ of\ substrate)}{Number\ of\ moles\ of\ catalyst}$$

$$= \frac{1.9695 \times 10 - 5}{2.5 \times\ 10\ - 4}$$

$$= 0.101$$

$$\text{TOF} = \frac{TON}{time(min)}$$

$$= \frac{TON}{time(min)}$$

$$= \frac{0.1012}{75}$$

$= 0.001349\ min^{-1}$

Similarly, TOF and TON of Ag@g-C_3N_4 containing $AgNO_3$ of different qualities can be calculated, as shown in Table 2
